# Supplementary material for: LEF1 Enhances the Progression of Colonic Adenocarcinoma via Remodeling the Cell Motility Associated Structures
Source: Int J Mol Sci. 2021 Oct 8;22(19):10870. doi: 10.3390/ijms221910870 (PMC8509209; doi:10.3390/ijms221910870)
Supplement: Supplementary file 1 [file ijms-22-10870-s001.zip › ijms-1384814-supplementary.pdf]

# Supplemental Materials

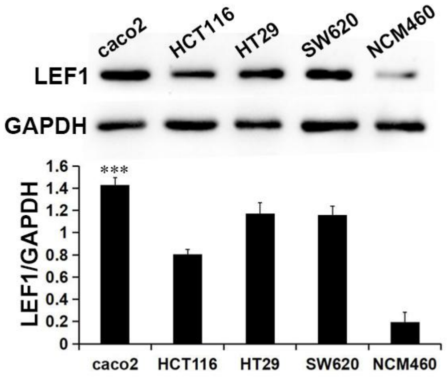


**Figure S1. LEF1 protein expressions in colon cancer cells and human intestinal epithelial cells.** ***$P < 0.01$.
